# Rare Form of Cardiac and Renal Disease, Difficult of Diagnosis

**Published:** 1874-04-15

**Authors:** N. S. Davis


### MEDICAL EXAMINER.

A

*Semi-Monthly Journal of Medical Sciences.*

EDITED BY N. S. DAVIS, M.D., AND F. H. DAVIS, M.D.

NO. VIII. CHICAGO, APRIL 15, 1874. VOL. XV.

### Original Communications.

### RARE FORM OF CARDIAC AND RENAL DISEASE, DIFFICULT OF DIAGNOSIS

REPORTED BY N. S. DAVIS, M.D.

MR. C., aged thirty-one years, a tin-smith, native of England, first presented himself to me for examination in the early part of the year 1873.

He presented a moderately anæmic look, though not emaciated; temperature natural; pulse unsteady, and sometimes intermittent, but slightly accelerated and soft; tongue clean; expression of countenance sad, or dejected; feet and whole lower extremities moderately swollen, from œdematous infiltration. He complained of general weakness, impaired appetite; shortness of breath, on attempting exercise; dull pain in the epigastric and right hypochondriac regions, with pain and sense of pressure directly over the pubes; bowels inactive; and urine less than natural, and slightly albuminous. The epigastric and left hypochondriac regions were full; but neither by percussion or palpation could I detect any well-defined tumor, or visceral enlargement. Percussion showed a moderately-increased area of cardiac dullness, but all other parts of the chest resonant as natural.

Auscultation revealed no cardiac valvular murmurs, and no pericardial friction sound, but a certain unsteadiness of rhythm and feebleness of impulse, with occasional intermission. Pulmonary sounds natural. The examination of the patient left a strong impression that the heart and kidneys were the principal organs involved in disease; and yet a close analysis of the symptoms left the nature of the disease unexplained in both. The urine was neither so scanty nor so highly albuminous as to correspond with the amount of œdema of the lower extremities, the fullness of the left half of the abdomen, and the occasional severe abdominal pains. The symptoms relating to the heart were still more unsatisfactory. The increased area of cardiac dullness, and unsteady rhythm of the heart, could not be from serous effusion into the pericardium, because the cardiac sounds and impulse were not distant from the surface, nor was the respiration hurried and oppressed when in the horizontal position. There was no increased impulse, sustained force of systolic contraction, or valvular murmurs, as in muscular hypertrophy; neither was there any loud ringing quality to the sounds, as in hypertrophy with dilatation of the cavities. Neither could the weak and irregular action of the heart be attributed either to fatty degeneration or to derangement of innervation, for these would not be accompanied by enlargement.

Hence, I was wholly unable to satisfy myself concerning the exact pathological condition of the heart. But believing there was some obscure organic change taking place in the kidneys, and, perhaps, mesenteric glands, I advised a mildly alterative and diuretic treatment, which resulted in an increase in the action of the kidneys, a disappearance of all traces of albumen from the urine, and a gradual removal of the œdema of the lower extremities. Still, he continued to complain of a deep-seated feeling of fullness and stiffness through the lumbar and left hypochondriac regions, with occasional sharp pains, and a sense of pressure just above and behind the symphisis pubis, and inability to endure more than very moderate exercise. The cardiac phenomena, also, remained unchanged.

He continued to call on me for advice occasionally through the summer, and was kept on light, plain food, mild tonics, especially such as were calculated to promote digestion and keep the bowels regular, occasional alteratives and diuretics, as the urine frequently became scanty, and moderate exercise in the open air. The general tone of his health improved gradually, until, for two months in the autumn, he felt confident that he would be able to resume his business in the spring.

Early in the winter, however, he came to me again, with the same obscure symptoms in the cardiac and abdominal regions, to which was added a subacute bronchitis, characterized by severe, harsh cough; soreness on both sides; shortness of breath; a scanty mucus expectoration; a copious mixture of moist and dry rhonchi; no increased dullness on percussion, and but little general fever. He was directed the following mixture, in doses of a teaspoonful four times a day, and a laxative pill to regulate the bowels:

℞.—Ammo. hydrochlo., ʒ iij.

Ant. et. pot. tart., grs. ij.

Morph. sulph., grs. iij.

Glycyrrhizæ syr., ℥ iv.—Mix.

His bronchial symptoms slowly disappeared; but they were renewed with considerable severity two or three times during the winter. The last of these attacks was about four weeks before his death, during which the breathing was much obstructed, and the expectoration and physical signs indicated slight pneumonic exudation.

Just as he recovered from this attack, a violent turn of vomiting and purging supervened, the discharges from the bowels being copious, thin, and frequent, and accompanied by severe abdominal pains. He was ordered the following mixture, in doses of a teaspoonful every hour, at first, and every two hours after the first three doses:

℞.—Acid carbolic cryst., grs. viij.
Glycerine, ℥ ss.
Tinct. opii et camph., ℥ iiss.
Aqua ℥ ij.—Mix.

At the end of twenty-four hours the vomiting had ceased; the intestinal discharges became less frequent, but mixed with mucus and blood.

Although a variety of remedies were faithfully used, yet they only served to lessen the frequency of the passages and the abdominal pains, but did not suppress the intestinal disease. The patient seemed to lose all appetite and power of assimilation, lost flesh rapidly, and died from exhaustion on the 18th of March, 1874. Twenty-four hours after death a *post-mortem* examination was made by Prof. J. S. Jewell, aided by Drs. F. H. Davis, and H. M. Bannister.

On opening the chest, the lungs and their appendages were apparently healthy; the pericardium was closely adherent to the surface of the heart, over most of its extent; but the whole organ was nearly double its natural size. The increase of bulk was owing, in part, to increase of muscular structure, and in part to enlargement of the ventricles. The pericardial adhesions appeared very old, and contained a thick layer of calcareous matter, making the heart appear as if almost completety encased in a layer of bone. The valves and endo-cardial surface of the heart appeared entirely natural. [The section presented is from the apex of the heart, and shows the thickness of the muscular structure, at the apex of the left ventricle—the pericardium exteriorly—and the calcareous deposit between the latter and the proper surface of the heart]. On opening the abdomen, no important morbid changes were noticed, except in the left kidney and ureter. The kidney was enlarged to nearly four times its natural size; of lighter color, especially in spots; regular in outline and feel; with no marks of recent or acute inflammation. The pelvis and ureter, to within one or two inches of the bladder, were greatly distended, the ureter being, in some places, more than an inch in diameter. This enlargement was apparently occasioned by a calculus impacted in the lower end of the enlarged part of the ureter, which may still be seen in the specimen presented to you. On laying open the kidney, the distended pelvis and infundibula presented the usual multilocular cystic appearance, and were filled with a turbid sero-purulent fluid, with numerous concretions varying in size from a pin's head to a large pea, and irregular in shape. The lining membrane of the ureter and pelvis of the kidney was thickened, and presented the usual white color seen in chronic pyelitis. But the increased bulk of the kidney was not altogether due to distention of the pelvis and infundibula. Both the cortical texture and pyramidal bodies were greatly hypertrophied or enlarged, and changed in appearance. Large portions presented a light gray or yellowish color, and the appearance of having undergone caseous or tuberculous degeneration. [All these changes are well represented in the section of the kidney here presented, and the dilated portion of the ureter. Some of the calculous concretions still adhere to one or two of the pouches embraced in the section, and others may be felt or seen in the ureter]. The mucous membrane of the ilium and colon was not examined; neither were the contents of the cranium. The chief object in reporting this case, aside from the interest attached to an examination of the morbid specimens, is to illustrate the difficulty of making a satisfactory diagnosis concerning the pathological condition of the heart. In the course of a somewhat extended practice, I have met with but one case previously, presenting similar difficulty of making an exact diagnosis; and after death there was the same combination of old pericardial adhesion, calcareous encasement, and hypertrophy of muscular structure, with perfect condition of the valves and endocardium.

